# Successful Surgical Ligation of a Giant Tarlov Cyst at the Cyst Neck Including the Nerve Root: A Case Report

**DOI:** 10.7759/cureus.81909

**Published:** 2025-04-08

**Authors:** Eitaro Okumura, Motoo Kubota, Ryo Hashimoto

**Affiliations:** 1 Spinal Surgery, Kameda Medical Center, Kamogawa, JPN

**Keywords:** cyst neck ligation, giant, intraoperative nerve monitoring, sacral perineural cyst, tarlov cysts

## Abstract

Perineural cysts are meningeal protrusions at nerve roots that can be asymptomatic but may require surgical intervention. Tarlov cysts, specifically located at sacral nerve roots, lack a consensus on optimal surgical treatment. This case report describes surgical management of a giant Tarlov cyst. A 29-year-old woman with infertility, low back pain, and constipation was found to have an 11 cm giant pelvic cyst originating from the left S3 nerve root. Preoperative imaging and symptoms suggested a perineural cyst. Intraoperative electromyographic monitoring revealed that the S2 nerve root could compensate for S3 function. Consequently, we performed radical ligation of the cyst neck together with the left S3 nerve root. Postoperatively, the patient's sensory disturbance in the posterior left thigh improved. Two months after surgery, the cyst remained regressed, constipation had improved, and the patient showed a favorable outcome. This case demonstrates successful surgical ligation of a giant Tarlov cyst, suggesting that when intraoperative nerve monitoring confirms compensatory nerve root function, radical nerve root ligation can be a viable surgical option.

## Introduction

Perineural cysts are meningeal protrusions occurring at nerve roots, which are often asymptomatic but may require surgical intervention when causing pain or radiculopathy. Specifically, Tarlov cysts are sacral perineural cysts arising between the peri and endoneurium of the posterior spinal nerve root at the dorsal root ganglion. These neurological formations occur with a global prevalence of approximately 4.27%, demonstrating a relatively uncommon occurrence. Interestingly, while these cysts are widespread, they remain predominantly asymptomatic, with only 1% of cases manifesting noticeable symptoms [1]. Demographically, these cysts show a distinct pattern, predominantly appearing in female patients within the age range of 50-60 years [1]. 

Despite their prevalence, the exact mechanism behind their formation remains unclear. Researchers have proposed multiple theoretical frameworks to explain their origin, including potential causes such as inflammatory processes, traumatic events, congenital predispositions, and degenerative changes [2,3]. The most currently accepted scientific explanation suggests that these cysts emerge from a disruption in the cerebrospinal fluid (CSF) and venous drainage mechanism, specifically at the junction between the perineurial and epineurial layers [4-6]. 

Tarlov cysts are most commonly diagnosed by lumbosacral magnetic resonance imaging (MRI) and can often be demonstrated by computed tomography (CT) myelography to communicate with the spinal subarachnoid space. The cyst can enlarge via a net inflow of CSF, eventually causing symptoms by distorting, compressing, or stretching adjacent nerve roots [7]. Patients' symptoms include radicular pain, sensory dysesthesias, urinary and/or bowel symptoms, and sexual dysfunction [1]. The most effective treatment of symptomatic Tarlov cysts, with options including non-surgical management, cyst aspiration and injection of fibrin glue, cyst fenestration, and nerve root imbrication, is debated [8]. Unfortunately, there is no consensus on patient selection or management approaches for symptomatic Tarlov cysts [9]. 

Given the ongoing debate about optimal management, we present a case of a giant Tarlov cyst successfully treated with surgical ligation.

## Case presentation

**Figure 1:**
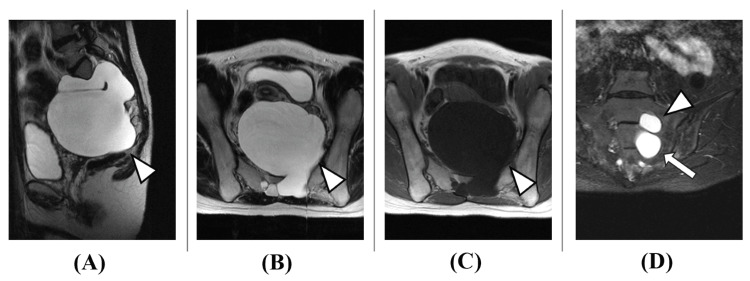
Initial pelvic MRI (A) T2 sagittal, (B) T2 axial, (C) T1 axial, (D) T2 coronal A cystic mass with a maximum diameter of 11 cm occupying the pelvic cavity was observed. The contents appeared as a uniform fluid collection with MRI T2 high intensity (arrowheads in A, B) and MRI T1 low intensity (arrowhead in C). A perineural cyst originating from the S2 (arrowhead in D) and S3 (arrow in D) nerve roots was suspected.

A 29-year-old woman presented with infertility, low back pain, and constipation. She had no significant medical history. Eight years earlier, lumbar MRI performed for low back pain evaluation had identified a cyst in the lumbar region. One year before presentation, a repeat lumbar MRI for infertility evaluation revealed a giant pelvic cyst. Surgical intervention was deemed necessary for her desire to conceive, leading to her referral to our hospital. At presentation, while there was no obvious lower limb weakness, she had numbness in the posterior left thigh and decreased pain sensation around the anus. Lumbar MRI showed a giant pelvic cyst with a maximum diameter of 11 cm, suggesting a meningeal cyst protruding from the left S2 and S3 nerve roots, indicating a possible perineural cyst (Figure 1).

**Figure 2:**
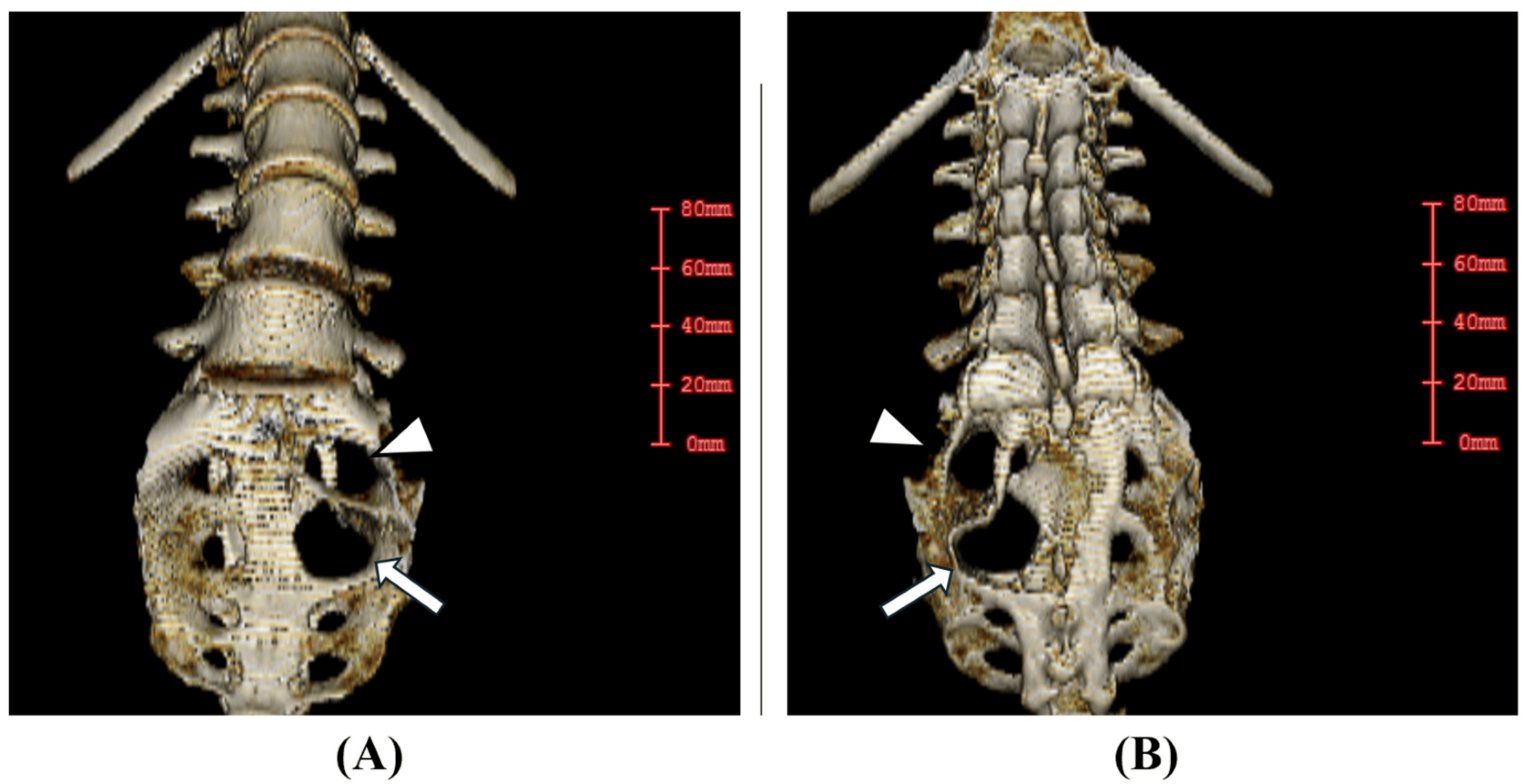
Initial pelvic CT Images (A) Lumbosacral 3D CT anterior view, (B) Lumbosacral 3D CT posterior view The cyst was large and associated with bone destruction of the left S2 foramen (arrowheads in A, B) and left S3 foramen (arrows in A, B).

The cyst was extremely large and had caused bone destruction of the left S2 and S3 foramina (Figure 2).

**Figure 3:**
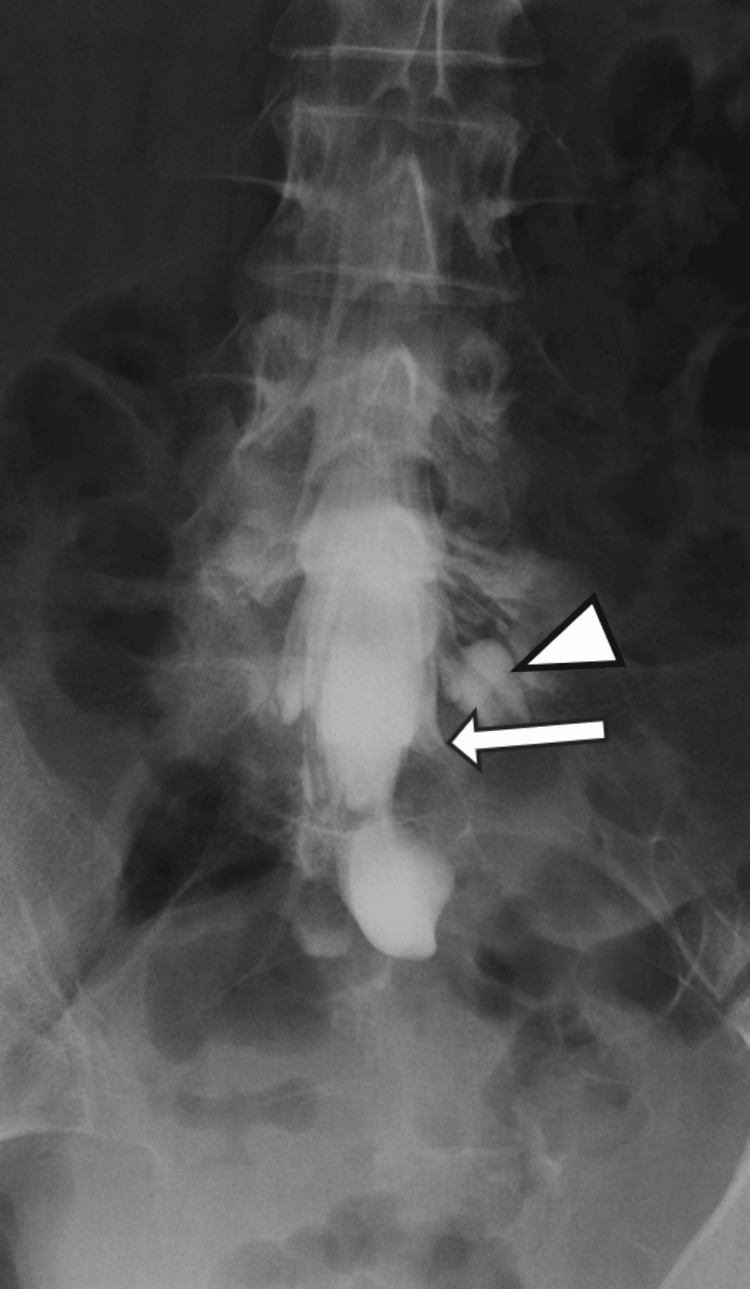
Initial myelography The left S1 nerve root (arrowhead) was well enhanced with contrast, the left S2 nerve root (arrow) was slightly enhanced, and the left S3 nerve root was not enhanced. This suggests that there is physical compression making it difficult for contrast medium to flow into the S2 nerve root and S3 nerve root. Additionally, there is minimal inflow of contrast medium into the cyst.

Myelography showed that while the left S2 nerve root was slightly enhanced with contrast, the left S3 nerve root was not enhanced, and there was minimal contrast medium flow into the cyst (Figure 3).

**Figure 4:**
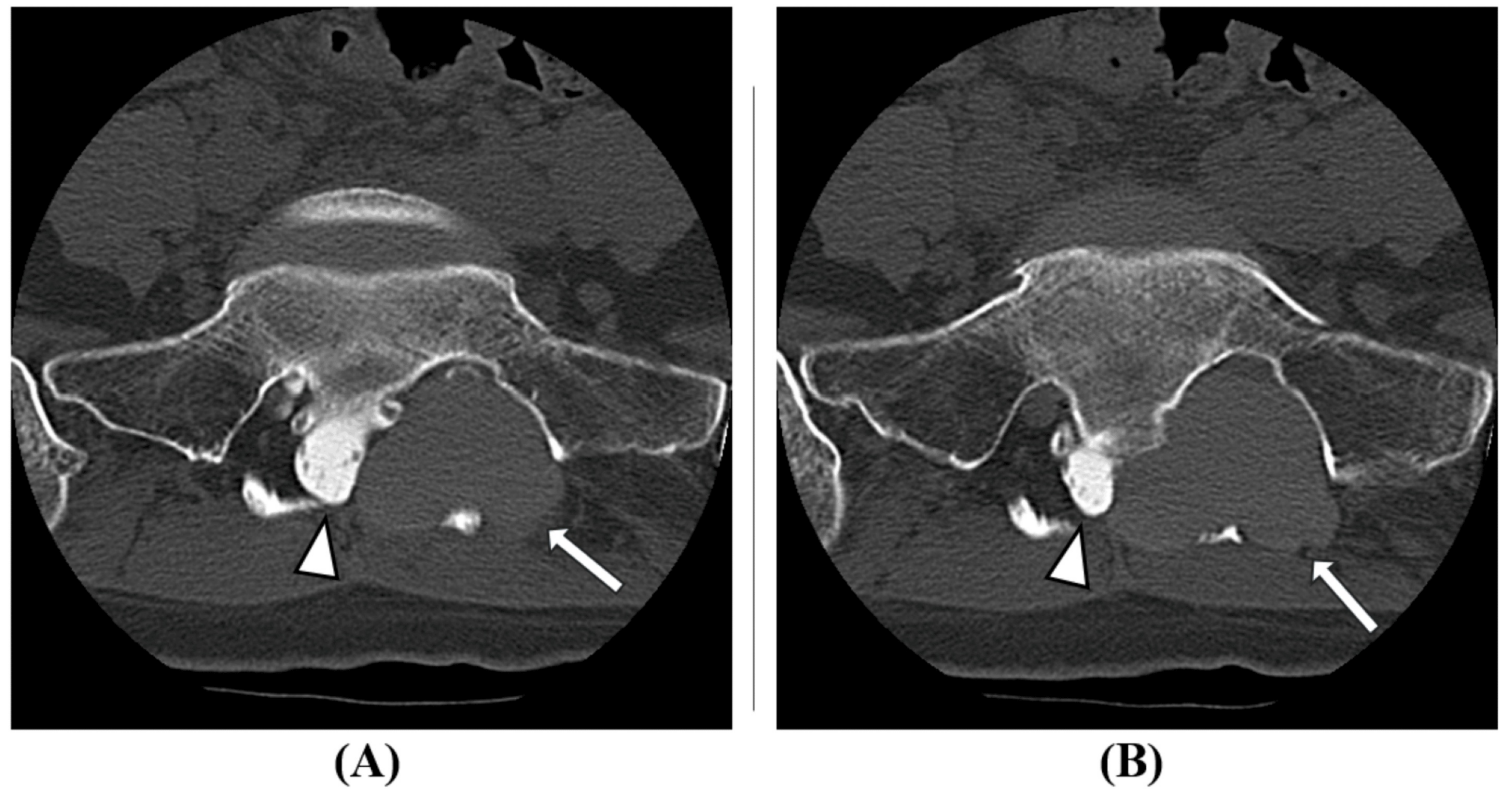
Post-myelography CT axial images (A) S2 level, (B) S3 level Good contrast enhancement is obtained within the spinal canal (arrowheads in A, B), but there is minimal inflow of contrast medium into the cyst (arrows in A, B). This suggested poor communication between the cyst and the subarachnoid space.

Post-myelography CT also showed poor inflow of contrast medium into the cyst (Figure 4).

**Figure 5:**
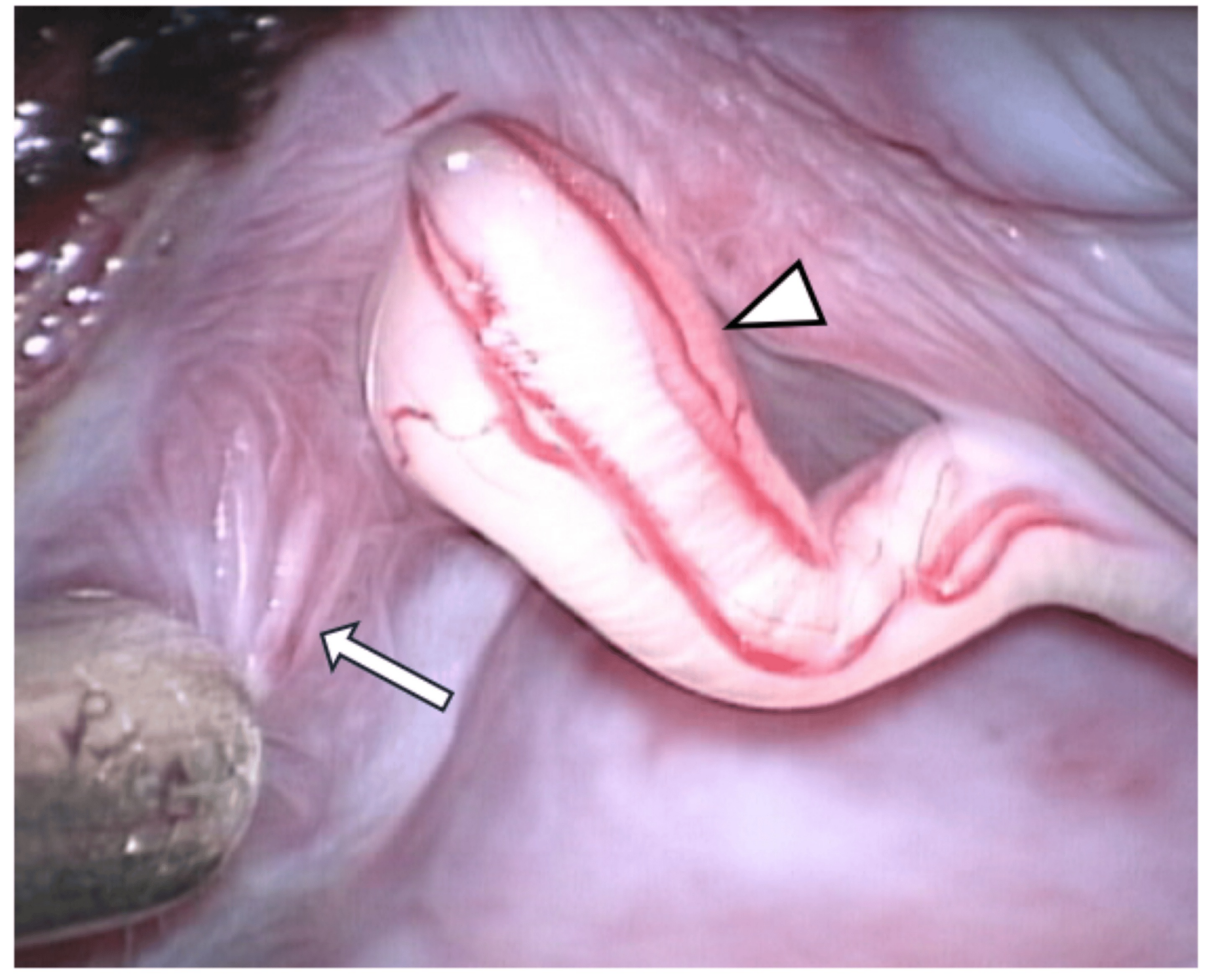
Intraoperative image after partial excision of the cyst wall The S3 nerve root was running through the cyst (arrowhead). Cerebrospinal fluid inflow was observed during the Valsalva maneuver. Neural fibers are suggested in part of the cyst wall (arrow).

Intraoperatively, the cyst was compressing the left S2 nerve root but this root was not involved in the cyst formation. The cyst originated solely from the left S3 nerve root, with the left S3 nerve root passing through the cyst (Figure 5). No continuous leakage of cerebrospinal fluid into the cyst was observed, but cerebrospinal fluid outflow into the cyst was seen during the Valsalva maneuver, suggesting that it was a Tarlov cyst with potential communication with the subarachnoid space.

**Figure 6:**
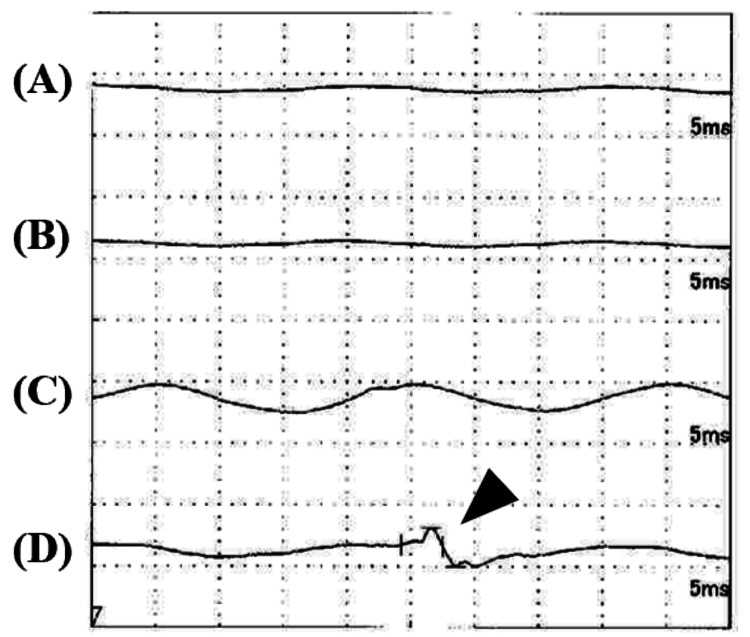
Electromyography Findings (A) Pudendal nerve, (B) Anal sphincter, (C) Lower limb muscles, (D) Dorsal interosseous muscles Intraoperative stimulation of the S3 nerve root at 1 mV yielded no response from the pudendal nerve, anal sphincter, or lower limb muscles. Only the interosseous muscles showed a response (black arrowhead). The S2 nerve root showed similar findings.

Intraoperative stimulation of the S2 and S3 nerve roots with electromyographic monitoring of the pudendal nerve, lower limb muscles, and anal sphincter showed interosseous muscle response from S3 but no response from the pudendal nerve or anal sphincter (Figure 6).

**Figure 7:**
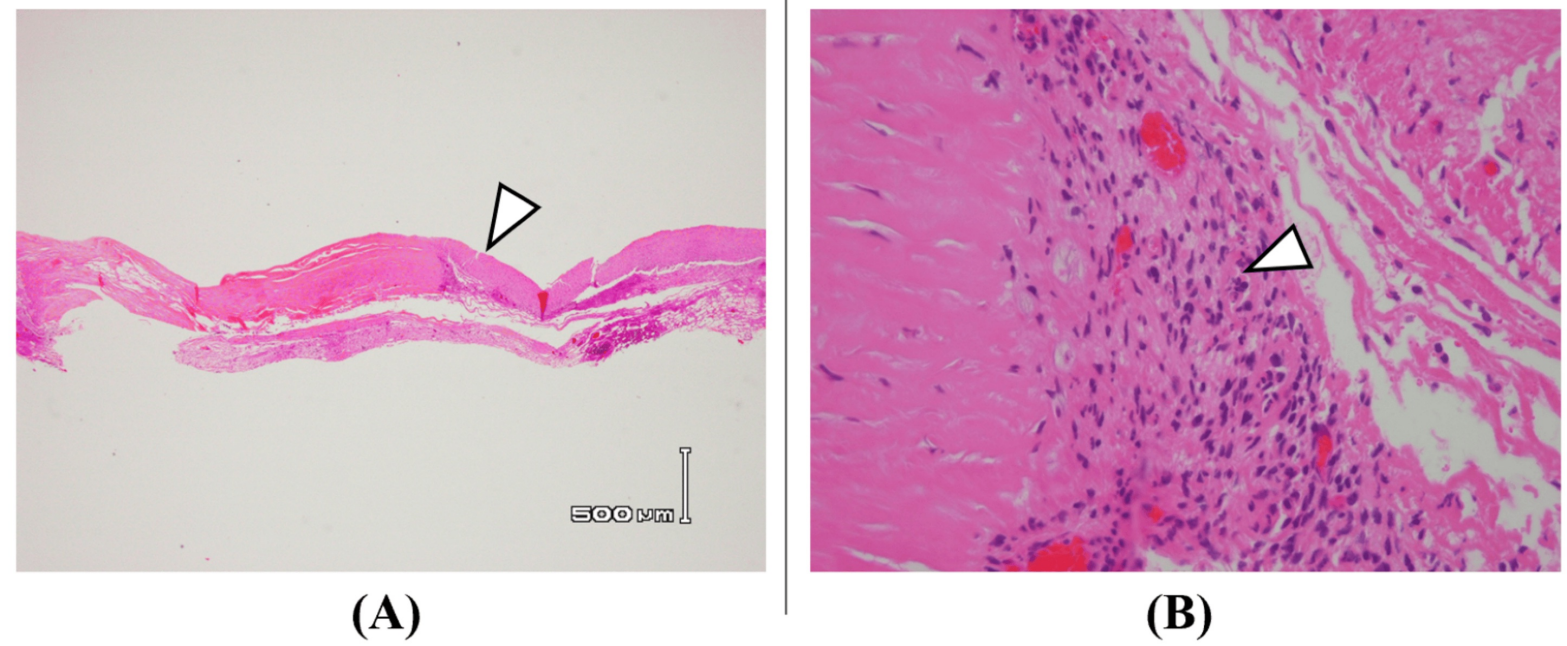
Histopathological findings (H&E staining) (A) 12.5x magnification, (B) 400x magnification Histopathological examination of the cyst wall showed primarily fibrous tissue with few cells (arrowhead in A) and some connective tissue containing blood vessels and inflammatory cells (arrowhead in B). The histopathological findings of Tarlov cyst walls most frequently include fibrous collagenous and membranous connective tissue, and inflammatory changes may sometimes be observed [10]; the pathological results in this case were consistent with these findings.

Since S2 also showed interosseous muscle response, we determined that S3 function could be compensated. Therefore, after partial cyst excision, we performed radical ligation of the cyst neck together with the left S3 nerve. Postoperatively, the sensory disturbance in the posterior left thigh improved. Sensation around the anus in the left S3 nerve distribution remained unchanged, suggesting dual innervation from S2. Histopathological examination of the cyst wall showed primarily fibrous tissue with few cells and some connective tissue containing blood vessels and inflammatory cells (Figure 7).

**Figure 8:**
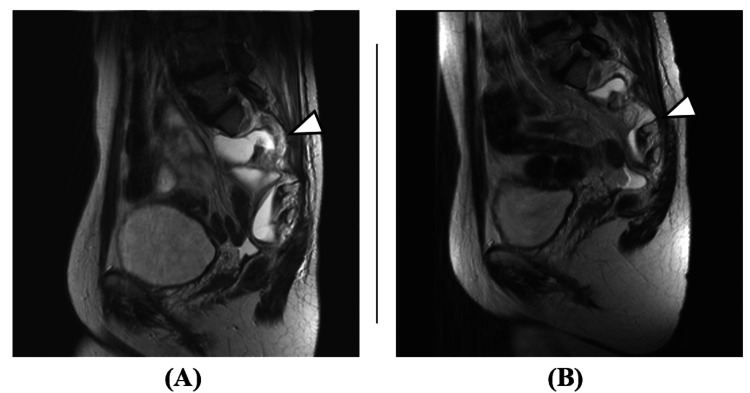
Postoperative pelvic MRI (T2 sagittal) (A) One week after surgery, (B) Two months after surgery The cyst wall (arrowheads) regressed over time.

Two months after surgery, the cyst remained regressed, constipation had improved, and the patient continued to show a favorable outcome (Figure 8). However, sacral bone destruction remains, and pain around the sacrum persists.

## Discussion

Klepinowski et al.'s meta-analysis of 22 imaging studies reported that Tarlov cysts are frequently located in the sacrum and occur in both single and multiple locations with a mean 11.9 mm (95%CI, 10.8-12.9 mm) cyst diameter [11]. While there are reports of giant Tarlov cysts exceeding 10 cm [1,12], the one in our case measured 11 cm × 10 cm × 9 cm, making it relatively large among reported Tarlov cyst cases. 

The progression and expansion of Tarlov cysts may result in distorting, compressing, or stretching nerves running through the cyst or compressing neighboring spinal nerve fibers, resulting in various neurological symptoms depending on the spinal level of involvement [13]. Cysts may also become sufficiently large to initiate bone tissue erosion causing irritation of periosteal pain fibers and, in rare cases, have been responsible for sacral insufficiency fractures [14]. In our case, the Tarlov cyst originated from S3 and was 11 cm in diameter, protruding from the S1/2 and S2/3 foramina to the anterior surface of the sacrum, accompanied by bone destruction (Figure 2). This likely contributed to the patient's lower back pain. Furthermore, since the Tarlov cyst was massive enough to occupy the pelvic cavity, it significantly compressed the intestines and female adnexa physically, which likely contributed to constipation and infertility. 

Nabors et al. described spinal meningeal cysts as diverticula of the spinal meningeal sac, nerve root sheath, or arachnoid [15]. For simplicity, they referred to all as meningeal cysts and introduced a simplified MRI-based classification for these conditions consisting of three groups: type I, extradural meningeal cysts without spinal nerve root fibers; type II, extradural meningeal cysts with spinal nerve root fibers; and type III, spinal intradural meningeal cysts. Our case can be classified as Nabor's type II. Surgical objectives for Tarlov cysts are generally to relieve nerve compression and/or stimulation, stop bone erosion, and relieve symptoms. There is no consensus on the optimal surgical method for Tarlov cysts, and there have been numerous evolving surgical techniques. Surgical procedures include sacral laminectomy, cyst fenestration, fenestration with CSF drainage, and identification and repair of the CSF fistula using fat and fibrin sealant, cyst neck ligation, partial cyst excision, nerve root imbrication, and abdominal surgery. Specifically, for Nabor's type I cysts without nerve roots inside, radical cyst neck ligation is often performed, while for Nabor's type II cysts with nerve roots inside, a combination of partial cyst excision and nerve root imbrication is considered [16,17]. In either case, intraoperative nerve monitoring is generally performed. In our case, intraoperative nerve monitoring was performed, and stimulation of the S2 and S3 nerve roots with electromyographic monitoring of the pudendal nerve, lower limb muscles, and anal sphincter showed interosseous muscle response from S3 but no response from the pudendal nerve or anal sphincter. Since S2 also showed interosseous muscle response, we determined that S3 function could be compensated. Therefore, instead of combining partial cyst excision with nerve root imbrication, we ligated the cyst neck together with the left S3 nerve to achieve a radical cure. As expected, there was no postoperative functional decline in the S3 region, presumably compensated by S2, and the patient had a favorable outcome. 

However, complications cannot be ignored. Kameda et al. reported an overall complication rate of 16.9% [18]. The main complications included CSF leaks 4.8%, surgical site infections 4.3%, and new or worsened bladder dysfunction 2.1% [18]. Kameda et al. also reported that complications were thought to be mainly related to the inadequate closure of the dura and/or handling sacral nerve roots. Therefore, it is essential to consider the optimal surgical technique for each case. In this case as well, if intraoperative monitoring had shown that the S3 nerve root function could not be compensated, alternative approaches such as partial cyst excision, cyst fenestration, and nerve root imbrication might have been selected.

## Conclusions

We reported a case of successful surgical ligation of a giant Tarlov cyst at the cyst neck together with the S3 nerve root, resulting in a favorable outcome. Intraoperative nerve monitoring plays an important role in deciding which surgical approach to take for Tarlov's cyst. Although this was a relatively large Tarlov cyst, if intraoperative nerve monitoring confirms that other nerve roots can compensate for the affected root, ligation of the cyst neck together with the nerve root inside the cyst should be considered. However, it should be noted that even if monitoring shows no issues, there is a risk of persistent sensory loss or bowel/bladder dysfunction postoperatively. It is important to continue to evaluate treatment outcomes with long-term follow-up. Careful patient selection and comprehensive neuroanatomical understanding can successfully manage even giant Tarlov cysts.
